# Clinical, Psychopathological, and Personality Characteristics Associated with ADHD among Individuals Seeking Treatment for Gambling Disorder

**DOI:** 10.1155/2015/965303

**Published:** 2015-07-01

**Authors:** N. Aymamí, S. Jiménez-Murcia, R. Granero, J. A. Ramos-Quiroga, F. Fernández-Aranda, L. Claes, A. Sauvaget, M. Grall-Bronnec, M. Gómez-Peña, L. G. Savvidou, A. B. Fagundo, A. del Pino-Gutierrez, L. Moragas, M. Casas, E. Penelo, J. M. Menchón

**Affiliations:** ^1^Department of Psychiatry, University Hospital of Bellvitge-IDIBELL, 08097 Barcelona, Spain; ^2^CIBER Fisiología de la Obesidad y Nutrición (CIBERobn), Instituto Salud Carlos III, 08907 Barcelona, Spain; ^3^Departament de Psicobiologia i Metodologia, Universitat Autònoma de Barcelona, 08193 Barcelona, Spain; ^4^Department of Psychiatry, University Hospital of Vall d'Hebron, 08035 Barcelona, Spain; ^5^CIBER Salud Mental (CIBERSAM), Instituto Salud Carlos III, 08907 Barcelona, Spain; ^6^Department of Psychiatry and Legal Medicine, Universitat Autònoma de Barcelona, 08193 Barcelona, Spain; ^7^Department of Psychology, Catholic University of Leuven, Tiensestraat 102, 3000 Leuven, Belgium; ^8^Department of Addictology and Psychiatry, University Hospital of Nantes, 44093 Nantes, France; ^9^Departament d'Infermeria de Salut Pública, Salut Mental i Maternoinfantil, Escola Universitària d'Infermeria, Universitat de Barcelona, 08907 Barcelona, Spain

## Abstract

*Objectives*. (1) To assess the current presence of ADHD symptoms among patients seeking treatment for gambling disorder; (2) to explore clinical and sociodemographic differences between patients who score high and low on the measure of ADHD symptoms; (3) to analyze whether the presence of ADHD symptoms is associated with more severe psychopathology and with specific personality traits; (4) to analyze the mediating role of ADHD symptoms in the relationship between novelty seeking and gambling severity. *Method*. A total of 354 consecutive patients were administered an extensive battery assessing gambling behavior, psychopathology, and personality traits. *Results*. Male and female gamblers did not differ significantly in their mean scores on the ADHD measure. However, younger participants aged 18–35 scored higher. Higher ADHD scores were also associated with greater severity of gambling disorder and more general psychopathology. Regarding personality traits, high persistence and self-directedness were negatively related to ADHD scores, while in women alone a positive correlation was found between ADHD scores and scores on harm avoidance and self-transcendence. *Conclusion*. The presence of ADHD symptoms in both male and female gambling disorder patients may act as an indicator of the severity of gambling, general psychopathology, and dysfunctional personality traits.

## 1. Introduction

Attention-deficit/hyperactivity disorder (ADHD) is an impairing condition that is mainly characterized by symptoms of impulsivity, hyperactivity, disorganization, and inattention [1]. This definition has been modified in the fifth edition of the* Diagnostic and Statistical Manual of Mental Disorders *(DSM-5) [2] to better describe the experience of affected adults. Although it is mostly associated with childhood, the symptoms may persist, with certain modifications and alternative expressions, into adult life, this being the case in up to 40% of cases [3–5]. Current prevalence rates for ADHD are in the range of 4-5% for adults and 6–9% for children [6, 7]. Studies have also shown that around 80% of individuals with ADHD present a comorbid psychiatric disorder [8].

ADHD has commonly been reported in several psychiatric conditions, including affective disorders, personality disorders, substance abuse disorders, eating disorders, impulse control and somatic disorders [9–12], both as current and/or lifetime condition [13]. In addition, several studies have reported positive associations between behavior, addictive disorders, and ADHD in children followed up into adolescence and early adulthood, including when compared with controls [14–18]. However, the comorbidity between ADHD and gambling disorder (GD) has rarely been investigated.

Several studies that did explore this association in the 1980s and 1990s suffered from a number of methodological pitfalls. For example, their findings were based on retrospective data analyses or the samples used were small [19–21]. However, a number of recent studies have provided more specific results. For instance, the longitudinal study by Breyer et al. [22], which explored the relationship between ADHD and gambling in a general population sample, found that those subjects who reported childhood ADHD symptoms persisting into adulthood presented a more severe gambling problem than those with nonpersistent or no ADHD symptoms.

Regarding the personality traits of GD patients and their association with ADHD symptoms, few studies have been conducted in adolescents and young adults [23] examining the relationship between problem gambling and impulsivity in adolescents aged 12–19 years and found that those with a history of problem gambling also reported a greater number of ADHD symptoms. More recently, Davtian et al. [24] compared personality traits between GD patients with and without ADHD. Although they found no significant differences in the impulsivity dimension, GD patients with ADHD were more likely to have lower self-esteem, lower self-discipline, and higher levels of emotional instability and stress predisposition. Mention should also be made of the meta-analysis conducted by Lipszyc and Schachar [25] of studies that used the stop signal task to assess inhibitory control in patients with various psychiatric disorders. The results confirmed that patients with ADHD showed deficits in inhibitory response, whereas in GD patients this response was less impaired or even normal. The authors also reported that the comorbidity of ADHD with other pathologies has different effects on inhibition, although they did not study the relationship between ADHD and GD.

The goal of the present study was fourfold: (1) to assess the current presence of ADHD symptoms among patients seeking treatment for GD; (2) to explore clinical and sociodemographic differences between patients who score high and low on the measure of ADHD symptoms; (3) to analyze whether the presence of ADHD symptoms is associated with more severe psychopathology and with specific personality traits; and (4) to analyze the mediating role of ADHD symptoms in the relationship between novelty seeking and gambling severity.

## 2. Method

### 2.1. Participants

The initial sample included 391 individuals referred consecutively for assessment and outpatient treatment. All were recruited exclusively in one specialized unit, which is part of the public health system (viz., Pathological Gambling Unit of the Psychiatry Department at the University Hospital of Bellvitge, in Barcelona, Spain). The diagnosis of gambling disorder was confirmed by administration of the Structured Clinical Interview for DSM-IV Axis I disorders [26]. Of this initial sample, 37 participants were excluded due to missing data in the main variables considered by the present study. The final sample of 354 individuals was predominantly male (89.5%), and a majority had only primary or no formal education (58.6%). Over half the participants were in employment (57.2%). Mean age was 42.2 years (SD = 13.1), and they had been gambling for a mean of 5.4 years (SD = 6.8). Enrolment in the study was between May 2010 and July 2011. All participants signed a written informed consent document and the study was approved by the ethics committee of all the institutions involved.

### 2.2. Instruments

Participants were administered a comprehensive assessment battery that measured gambling behaviors, GD symptoms, ADHD symptoms, general psychopathology, and personality traits. The battery included internationally applied instruments in the GD field, namely, the South Oaks Gambling Screen (SOGS; [27]) and Stinchfield's Diagnostic Questionnaire for Pathological Gambling according to DSM-IV criteria [28], as well as the short screener version of the Adult ADHD Self-Report Scale [29], the Symptom Checklist-90-Revised (SCL-90-R; [30]), and the Temperament and Character Inventory-Revised (TCI-R; [31]). The use of two instruments for the identification of a GD diagnosis was due to the characteristics of each questionnaire. The SOGS establishes whether the individual is a probable case of GD or not and offers a wide range and useful information about the gambling behavior from a clinical point of view. Differently, the DSM-IV questionnaire provides the number of criteria met by the patient, and thus it permits us to infer the severity of the gambling behavior.

#### 2.2.1. South Oaks Gambling Screen (SOGS) [27]

The SOGS includes 20 items that yield a total score ranging from 0 to 20, with a score of five or more indicating a probable GD. The psychometric properties of the Spanish version of this questionnaire have been shown to be satisfactory: test-retest reliability was 0.98, internal consistency 0.94, and convergent validity 0.92 [32]. Several studies have also reported satisfactory psychometric properties for the SOGS as a measure of gambling severity in both clinical and general population samples [33–35].

#### 2.2.2. Stinchfield's Diagnostic Questionnaire for Pathological Gambling, according to DSM-IV Criteria [28]

This 19-item questionnaire assesses the ten DSM-IV [36] diagnostic criteria for pathological gambling and has shown satisfactory psychometric properties. Internal consistency, based on Cronbach's alpha, was .81 for the general population and .77 for a gambling treatment group. Convergent validity, estimated by the correlation with the SOGS, was .77 for a general population sample and .75 for a gambling treatment sample. This scale has been adapted for the Spanish population by [37]. Cronbach's alpha in the present sample was very good (*α* = .90).

#### 2.2.3. Adult ADHD Self-Report Scale (ASRS-v1.1) [29]

The ASRS-v1.1 is a self-administered scale designed to screen for ADHD in adults [29]. Examination of its psychometric properties has revealed good validity and reliability [38]. ASRS-v1.1 comprises the 6 out of 18 most predictive items of the Adult ADHD Self-Report Scale (ASRS) [39], Spanish validation by Ramos-Quiroga et al. [40], which was the measure used in the present study. A notable score on at least four of these six screener items is suggestive of ADHD [29]. Each item is responded to on a 5-point Likert scale from 0 = never to 4 = very often, such that the possible total score ranges between 0 and 24, with the cut-off being set at 12.

#### 2.2.4. Symptom Checklist-90-Revised (SCL-90-R) [30]

The SCL-90-R measures a broad range of psychological and psychiatric symptomatology. The questionnaire contains 90 items and measures nine primary symptom dimensions: somatization, obsession-compulsion, interpersonal sensitivity, depression, anxiety, hostility, phobic anxiety, paranoid ideation, and psychoticism. When scored it yields three global indices: the global severity index (GSI), designed to measure overall psychological distress; the positive symptom distress index (PSDI), designed to measure symptom intensity; and the positive symptom total (PST), which reflects the number of self-reported symptoms. The GSI can be used as a summary of the subscales. The SCL-90-R has demonstrated satisfactory psychometric properties in a Spanish sample, with mean internal consistency of .75 (*α* coefficient) [30, 41].

#### 2.2.5. Temperament and Character Inventory-Revised (TCI-R) [31]

This is a 240-item questionnaire with 5-point Likert response options [42]. It measures seven dimensions of personality: four of temperament (harm avoidance, novelty seeking, reward dependence, and persistence) and three character dimensions (self-directedness, cooperativeness, and self-transcendence). The Spanish version of the inventory has demonstrated satisfactory psychometric properties [43].

Additional demographic, clinical, and social/familial variables related to gambling were collected via a semistructured clinical interview described elsewhere [44].

### 2.3. Procedure

In accordance with the manualized assessment protocol and treatment model of the unit [45], participation in the study involved two visits. On the first visit, participants took part in a semistructured, face-to-face interview in order to further explore their gambling behavior, psychopathology, and personality traits. Sociodemographic data (e.g., education, occupation, and marital status) and required additional clinical information was also collected during this visit. On the second visit, participants completed all the remaining instruments mentioned above. The two interviews were conducted within a time-frame of one week by a clinical psychologist and a psychiatrist.

## 3. Statistical Analysis

Statistical analyses were carried out with SPSS20 for Windows. Indices of ADHD (prevalence rates for high ADHD score and means for the total ADHD score) were obtained and compared by gender, age, and main types of gambling (slot machines versus others). Prevalence rates were compared using the chi-square test, and prevalence ratios were estimated in order to assess the effect size of differences. Means for the ADHD-total scores were compared by means of analysis of variance (ANOVA), with post hoc comparisons (least significant difference procedure, LSD) being used to estimate the effect size of differences.

Patients who scored high and low on ADHD were then compared with respect to sociodemographic and clinical characteristics, applying chi-square tests (for categorical variables) and Student's *t*-test (quantitative variables). Cohen's *d* coefficient was calculated as a measure of effect size for the proportions and mean comparisons (effect size was interpreted as moderate for |*d*| > 0.50 and high for |*d*| > 0.80).

Multiple linear regressions were then used to explore the association between the ADHD-total score and the severity of GD (SOGS-total score and DSM-total score), general psychopathology (SCL-90-R scores), and personality profiles (TCI-R scores). These models are included as the independent variable the patients' score on each questionnaire scale and as the dependent variable the ADHD-total score. Five independent models (all adjusted by the patients' sex and age) were obtained and interpreted, namely, for the criterion SOGS-total score, the total number of DSM-IV criteria, the SCL-90-R first-order scales, the SCL-90-R second-order scales (PST, GSI, and PSDI), and the TCI-R scales. Each regression model included the interaction of questionnaire score with gender and age in order to examine the potential moderator effect of these two variables. Main effects were estimated and interpreted for nonsignificant interaction (*p* > .05) and single effects for significant interactions.

Finally, a structural equation model was developed (using Stata13 for Windows) to assess the possible mediating role of the ADHD-total score in the relationship between novelty seeking (measured by the TCI-R NS score) and gambling severity (DSM-total score). Goodness-of-fit was determined according to the following statistical criteria: nonsignificant result (*p* > .05) in the global chi-square test, root mean square error of approximation (RMSEA) lower than .08, comparative fit index (CFI) and the Tucker-Lewis index (TLI) higher than .90, and the size of residuals (standardized root mean square residual, SRMR) limited to .08 [46]. The overall predictive capacity was measured with the coefficient of determination (CD).

## 4. Results

### 4.1. Distribution of ADHD Scores: Prevalence and Mean Estimations

Table 1 shows the prevalence and mean scores for ADHD, taking into account the whole sample and stratifying by gender and age. No gender differences were observed in the proportion of participants with high ADHD scores (prevalence ratio [PR] = 1.47, *p* = .158), nor in the mean ADHD scores (mean difference [MD] = 0.78; *p* = .445).

Following Petry [47], patients were classified into three groups according to age upon entering treatment at the pathological gambling unit: young adults (age 18–35 years; *n* = 124), middle-aged adults (36–55 years; *n* = 166), and older adults (56–80 years; *n* = 64). The prevalence of participants with high ADHD scores differed between age groups (*χ* ^2^ = 7.28, df = 2, *p* = .026), with high scores being more common among young as compared with older adults (PR = 2.18, *p* = .013). Mean scores on the ADHD measure also differed according to age (*F* = 6.31, df = 2–351, *p* = .002): while the mean score of the younger participants (aged 18–35) was equal to that of the 36–55 age group (MD = 0.90; *p* = .105), the mean score of this group differed from that of the old-age participants (MD = 1.65; *p* = .017).

Finally, participants whose main problem was slot machines did not differ from those with other types of gambling problem (MD = 0.60; 95% CI [−0.86; 2.06]; *p* = .420; PR = 1.12; 95% CI [0.66; 1.90]; *p* = .679).

### 4.2. Comparison of Sociodemographic and Clinical Variables between Patients with High versus Low ADHD Scores

The first column of Table 2 gives the distribution of the main study variables for the whole sample, while the following two columns show the comparison between patients who obtained low and high ADHD scores, respectively. These two groups differed significantly in the percentage of employed patients (higher in the ADHD-low score group), the presence of comorbid disorders (higher prevalence in the ADHD-high score group for present and past comorbid disorders and family psychiatric history), the presence of alcohol abuse and other substance abuses (higher prevalence in the ADHD-high score group), and age (lower mean age in the ADHD-high score group). Effect sizes for differences in proportions and means were, however, in the moderate to low range.

### 4.3. Association between ADHD Total Score and Clinical and Personality Measures

Table 3 shows the association between ADHD scores and the level of gambling (SOGS-total score and DSM-total score), the level of general psychopathology (SCL-90-R scores), and personality traits (TCI-R scores). A positive association, independent of age and gender, was found between the level of gambling disorder and the level of ADHD (the higher the level of gambling the higher the ADHD score) (SOGS:* B* = 0.34, *p* < .001; DSM-IV criteria:* B* = 0.64, *p* < .001). Regarding the level of psychopathology, higher ADHD scores were positively associated with higher SCL obsessive-compulsive symptom scores (*B* = 0.29; *p* < .001) and also with higher SCL GSI scores, although the latter relationship was only significant for middle-aged (40 years old:* B* = 2.83; *p* = .035) and older patients (60 years old:* B* = 4.22, *p* = .005). Finally, the TCI-R dimensions harm avoidance, in females (*B* = 0.11; *p* = .005), and self-transcendence (*B* = 0.07; *p* < .001) were positively related to ADHD scores (higher scores on these TCI-R scales corresponded to higher levels of ADHD), whereas persistence (*B* = −0.03; *p* = .008) and self-directedness (*B* = −0.05; *p* < .001) were negatively associated with ADHD scores (high scores on these TCI-R scales corresponded to fewer ADHD symptoms).

### 4.4. Mediating Role of ADHD Symptoms in the Relationship between Novelty Seeking and Gambling Severity

**Figure 1:**
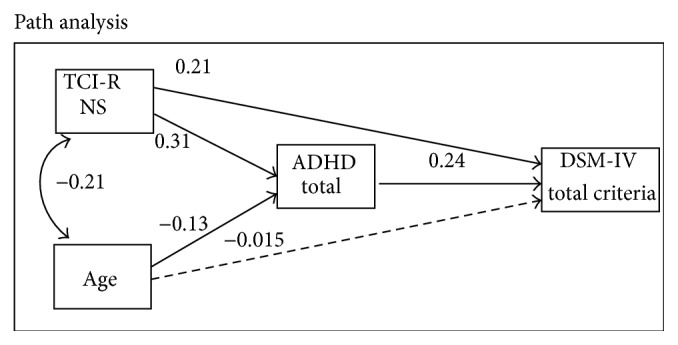
Structural equation model for novelty seeking, ADHD, and gambling severity. Results adjusted by patients' sex.

Figure 1 shows the path diagram and the standardized coefficients of the SEM developed to evaluate the mediating role of the ADHD total score in the relationship between novelty seeking (TCI-R NS-score) and gambling severity (DSM-total criteria). Due to the strong association between patients' age and the variables considered in the analysis, the former variable was considered as another predictor in the SEM. Results were also adjusted by patients' age. The SEM showed a very good fit: *χ* ^2^ = 2.28 (*p* = .131), RMSEA = .063, CFI = .986, TLI = .900, and SRMR = .018. The global predictive capacity was also good (CD = .166).

Novelty seeking showed a direct effect on gambling severity (*B* = 0.306, SE = .050, *z* = 6.10, and *p* < .001), as well as an indirect effect mediated through the ADHD-total score: high novelty seeking scores were predictive of high ADHD scores (*B* = 0.213, SE = .053, *z* = 4.00, and *p* < .001), and high ADHD scores were related to high gambling severity (*B* = 0.242, SE = .051, *z* = 4.79, and *p* = .001). Patients' age was negatively related to ADHD symptoms (the youngest patients obtained the highest ADHD scores; *B* = −0.128, SE = .054, *z* = −2.37, and *p* = .018), but age did not show a direct effect in relation to the DSM-total criteria (*B* = −0.015, SE = .053, *z* = −0.28, and *p* = .777). The path diagram shows that the ADHD-total score mediated the relationship between novelty seeking and gambling severity, as well as the relationship between age and gambling score.

## 5. Discussion

This study assessed the prevalence of ADHD symptoms in a sample of treatment-seeking GD patients and explored clinical and sociodemographic differences between patients who scored high and low on the ADHD measure, on psychopathology, and on certain personality traits. The meditating role of ADHD symptoms and the relationship between novelty seeking and gambling severity was also examined.

One of the main findings of the study was that the prevalence of ADHD symptoms in a consecutive clinical sample of treatment-seeking GD individuals was 23.2%. This result is in agreement with other studies that describe an association between the presence of ADHD and gambling problems [4, 21, 48, 49], with similar rates being obtained in both clinical and community samples, which might suggest the existence of shared vulnerabilities [50]. Interestingly, our analysis revealed no gender differences. Given that previous studies have reported higher rates of ADHD among males [51–55], we expected to find a smaller proportion of female patients with symptoms of ADHD. Our result can be interpreted in the context of research indicating that ADHD is a factor associated with GD regardless of sex [4, 48, 56].

Also in agreement with previous studies [22], younger patients have scored significantly higher on ADHD symptoms, a finding that supports the claim of Derevensky et al. [23] that adolescents with gambling problems frequently present more symptoms of ADHD. We also found that patients with ADHD symptoms reported a younger age of onset of gambling behavior, a result that is consistent with other recent studies [50]. This finding is particularly important given that early age of onset has been associated with greater severity of gambling behavior and more severe consequences in all areas of the individual's life [57–59]. In this context, LaBrie et al. [60] observed a particular vulnerability profile among adolescents and young people that was characterized by gambling behavior, regular consumption of alcohol, and episodes of binge drinking. Although a recent study by Ostojic et al. [61] found no relationship between childhood ADHD and increased risk of substance use in a clinical sample of young men, their results did suggest an association between these two conditions and being female, as well as with early gambling behavior. Our analysis of sociodemographic variables also showed higher rates of employment in the ADHD-low score group, a result that is consistent with studies reporting a close relationship between the severity of ADHD in GD and financial, employment, and even legal consequences [62, 63].

With respect to clinical variables, comorbid disorders and the abuse of alcohol and other substances (both current and lifetime) were all more common among patients with high ADHD scores. Previous studies have similarly found an association between adult ADHD and substance abuse [64, 65], impulse control disorders [21], eating disorders [12, 66], and bipolar disorder and borderline personality disorder [67, 68]. Regarding the predictive capacity of ADHD symptoms in relation to gambling behavior, psychopathology, and personality traits, our results showed an association between ADHD symptoms and severity of gambling behavior. Several authors, such as Breyer et al. [22] or Grall-Bronnec et al. [48], have also described a relationship between severity of gambling problems and the presence of ADHD. As for psychopathology (measured by means of the SCL-90-R), the presence of ADHD symptoms in our patients was positively associated with scores on the obsessive-compulsive dimension and the global severity index, although only for middle-aged or older GD patients. Previous studies have likewise reported an association between the presence of ADHD symptoms in GD and greater psychiatric comorbidity (affective and anxiety disorders, risk of suicide, and substance consumption), both in adults [48] and in adolescents [4, 23, 56]. However, it should be noted that some recent research failed to identify sociodemographic differences (gender, age, and education) or comorbidity related to ADHD among individuals with GD [50].

Regarding personality traits, our results showed that high scores on harm avoidance were related to ADHD, although only in women, whereas the positive association between ADHD and the character trait self-transcendence was observed for both sexes. Several studies have also described the association between ADHD and specific personality traits such as neuroticism [69] anxiety and negative affect [70] or harm avoidance as measured with the TCI-R [71]. Furthermore, Faraone et al. [72] reported the existence of elevated self-transcendence mean scores in samples of adults with ADHD. Therefore, the results obtained in the present study are in concordance with the previously described positive association between ADHD and dysfunctional personality profiles. Unlike the temperament dimensions (e.g., harm avoidance), which are stable over the lifespan given the inherited genetic basis, character traits (such as self-transcendence-ST) are strongly influenced by learning and the environment [73]. Individuals with high self-transcendence are potentially more spiritual, unpretentious, humble, judicious, insightful, and acquiescent. These traits may be advantageous when the person must confront problems and illnesses [74]. Considering that ADHD usually appears before the age of 12 and that, from childhood, people affected by this condition are likely to have suffered many problems in all areas of their lives (school, family, social, etc.). It could therefore be hypothesized that these character traits (viz., ST) have been learned and modulated progressively, as an adaptive mechanism to cope with the adversities that they have experienced throughout life, which are more prevalent during adulthood. This hypothesis would be consistent with the findings of Faraone et al., [72] who obtained this association in adults with ADHD. Differently, Cho et al., [75], in a sample of adolescents with another behavioral addiction (problematic internet use) and ADHD, observed that the patients showed low levels in these personality dimensions. The results obtained by Hee et al. [76] are also incongruent. These authors did not find differences in ST traits when comparing a group of children between 9 and 14 years with ADHD to a control group.

The analysis also revealed that high levels of persistence and self-directedness were negatively associated with the presence of ADHD. Davtian et al. [24] also compared two groups of individuals with GD, with and without associated ADHD. Although they found no differences in impulsivity, the group with ADHD symptoms presented higher levels of neuroticism, being more prone to anxiety, worry, depression, social isolation, and low self-esteem. These authors suggested that gambling behavior could be a maladaptive mechanism for regulating negative emotional states and stress. Although this hypothesis has received consistent support in certain subtypes of GD individuals [77–82], the findings of Davtian et al. [24] demonstrate that GD patients with comorbid ADHD presented emotional regulation problems, which is also in agreement with our findings (especially in women). It should also be noted that we observed no differences in the novelty seeking dimension, which measures, among other features, the impulsivity of the individual. In this regard, the increased levels of fantasy and imagination and the tendency to disconnect from the environment reported by these authors are likewise consistent with our results.

The study by Davtian et al. [24] also found that subjects with ADHD and GD showed low levels of conscientiousness and self-Discipline, that is, low self-esteem, lack of ambition, insecurity, low self-confidence, irresponsibility, and deficits in the ability to plan and organize life goals and objectives and low persistence to complete tasks. All these characteristics are similar to the persistence and self-Directedness dimensions measured using the Cloninger inventory in the present study.

Finally, the path diagram analysis indicates that ADHD symptoms mediate the relationship between novelty seeking and severity of gambling behavior, as well as the relationship between the latter and age. These results support the findings of previous studies showing a direct relationship between ADHD and excitement-seeking [24]. The connection between GD and impulsiveness could also be mediated by ADHD symptoms [22]. In turn, this biological and moderately inherited temperament trait, namely novelty seeking, [73] and younger age are associated with both ADHD symptoms [83].

This study has several limitations that need to be considered when interpreting the findings. First, the sample comprised treatment-seeking GD patients and therefore the results might not be generalizable to individuals with a GD who do not seek treatment. Second, the assessment procedures used did not allow for in-depth evaluation of comorbid disorders. Third, the cross-sectional design prevents us from establishing the direction of causality among the variables assessed. Fourth, the frequency of adult ADHD symptoms was estimated using a self-report screening instrument, which might explain the elevated prevalence of ADHD symptomatology in the study sample. Related to this limitation is the fact that the presence of childhood ADHD was not assessed, which would have been a prerequisite for an ADHD diagnosis in adulthood. Nevertheless, the ASRS has good psychometric properties to screen ADHD in adults, also in patients with comorbidities (in the present study, psychopathology and emotional distress were assessed by means of the SCL-90-R). Personality functioning was also based on self-report measures. While acknowledging these limitations, it is noteworthy that the study gathered information from a relatively broad clinical sample formed by consecutive treatment-seeking individuals with a GD and a path model was developed to describe the associations between sociodemographic characteristics, personality, ADHD, and GD severity.

## 6. Conclusions

The presence of ADHD symptoms in gambling disorder patients could be an indicator of the severity of gambling, general psychopathology, and dysfunctional personality traits. Furthermore, the findings of the path model suggest that both ADHD and GD severity are driven by high levels of novelty seeking and young age. Intervention programs should focus on training in self-control strategies and skills for dealing with negative emotions, on ways of increasing tolerance to boredom and maintaining persistence, on improving planning skills and learning to delay both responses and rewards, and on encouraging alternative thought and decision-making processes. Moreover, these results confirm the importance of developing preventive and early detection strategies for GD in adolescence since, in this stage of life, self-control, and self-regulation skills have not yet been consolidated and teens have a higher risk and greater vulnerability to being exposed to activities such as gambling.

## Figures and Tables

**Table 1:** Estimated prevalence of high ADHD scores and mean ADHD total score.

	High ADHD score: prevalence		ADHD total score: means		
	Prevalence	95% CI (prev.)	Mean	SD	95% CI (mean)
All sample (*N* = 354)	23.2%	19.1–27.8%	9.14	4.75	8.64–9.63

Sex					
Male (*N* = 317)	22.1%	17.9–27.0%	9.06	4.59	8.55–9.56
Female (*N* = 37)	32.4%	19.6–48.5%	9.84	5.95	7.85–11.82

Age groups (years)					
18–35 (*N* = 124)	30.6%	23.2–39.2%	10.02	4.73	9.18–10.87
36–55 (*N* = 166)	21.1%	15.6–27.9%	9.12	4.42	8.44–9.80
56–80 (*N* = 64)	14.1%	7.57–24.6%	7.47	5.18	6.18–8.76

**Table 2:** Comparison of patients based on high versus low ADHD score.

	Total sample (*N* = 354)	ADHD-low (*N* = 272)	ADHD-high (*N* = 82)	*p*	Cohen's |*d*|
Gender; %					
Males	89.5%	90.8%	85.4%	.158	0.17
Females	10.5%	9.2%	14.6%		

Education level; %					
Secondary or university	41.3%	41.4%	41.0%	.947	0.01
Primary or less	58.6%	58.6%	59.0%		

Marital status; %					
Married	49.8%	52.3%	41.8%	.102	0.21
Single-divorced-widowed	50.2%	47.7%	58.2%		

Employment status (employed); %	57.2%	60.1%	47.5%	**.046**	0.25

Other comorbid disorders at present; %	25.4%	20.0%	45.5%	**<.001**	**0.56**
Other comorbid disorders in past; %	39.8%	34.3%	60.4%	**.001**	**0.54**
Psychiatric disorders in family; %	58.6%	54.8%	73.1%	**.017**	0.39

Smokers; %	66.3%	66.7%	64.7%	.792	0.04
Alcohol abuse; %	7.6%	5.5%	15.4%	**.017**	0.33
Abuse of other substances; %	6.7%	4.5%	15.1%	**.006**	0.36
Other addictions	9.9%	9.5%	11.3%	.693	0.06

Main problem (reason for consultation): gambling; %	86.7%	87.1%	85.4%	.679	0.05
Types of gambling:					
Machines; %	84.4%	84.3%	84.5%	.975	0.00
Bingo; %	9.7%	10.3%	7.3%	.495	0.11
Lotteries; %	11.6%	12.3%	9.1%	.508	0.10
Casino; %	8.9%	9.4%	7.3%	.630	0.08
Playing cards; %	5.4%	5.9%	3.6%	.509	0.11
Bets; %	2.7%	2.5%	3.6%	.635	0.07
Internet; %	5.8%	4.9%	9.1%	.242	0.16
Others; %	1.9%	1.5%	3.6%	.306	0.14
Total number of gambling types; mean (SD)	1.33 (0.66)	1.33 (0.67)	1.26 (0.64)	.482	0.11

Age (years); mean (SD)	42.2 (13.13)	43.28 (13.08)	38.62 (12.76)	**.005**	0.36
Age at onset of gambling (years); mean (SD)	35.9 (13.93)	36.59 (13.97)	33.32 (13.61)	.152	0.24
Time since onset of gambling (years); mean (SD)	5.43 (6.77)	5.38 (6.75)	5.62 (6.94)	.834	0.03

Maximum money spent/episode (euro); mean (SD)	1205 (2747)	1154 (2687)	1402 (2989)	.582	0.09

Average money spent/episode (euro); mean (SD)	118 (256)	120 (242)	111 (304)	.844	0.03
Total accumulated debts (euro); mean (SD)	7908 (19986)	7566 (18951)	9351 (24076)	.604	0.08

Own income/month (euro); mean (SD)	1175 (690)	1203 (649)	1080 (808)	.188	0.17
Family income/month (euro); mean (SD)	2071 (1069)	2114 (982)	1923 (1328)	.266	0.16

SD: standard deviation. Bold: significant comparison (.05 level). Bold: moderate (|*d*| > 0.5) to high (|*d*| > 0.8) effect size.

**Table 3:** Association between gambling severity (SOGS and DSM total scores), measures of psychopathology (SCL-90-R) and personality (TCI-R), and ADHD scores.

	Interaction (*p* value)		Parameters for the predictor adjusted by gender and age					
	IV × sex	IV × age	SinEff	*B*	SE	Beta	*p*	95% CI (*B*)
Model for SOGS-total score	.965	.942		0.339	0.078	0.234	<.001	0.19; 0.49

Model for the number of DSM-IV criteria	.490	.454		0.635	0.111	0.298	<.001	0.42; 0.85

Model for SCL-90-R 1st-order scales								
SCL: somatization	.560	.419		−0.026	0.035	−0.055	.455	−0.10; 0.04
SCL: obsessive-compulsive	.171	.232		0.293	0.046	0.521	<.001	0.20; 0.38
SCL: interpersonal sensitivity	.388	.941		0.060	0.055	0.100	.279	−0.05; 0.17
SCL: depressive	.140	.413		−0.028	0.035	−0.073	.422	−0.10; 0.04
SCL: anxiety	.721	.168		0.038	0.062	0.070	.537	−0.08; 0.16
SCL: hostility	.564	.377		0.017	0.059	0.019	.778	−0.10; 0.13
SCL: phobic anxiety	.209	.565		0.025	0.067	0.027	.706	−0.11; 0.16
SCL: paranoid ideation	.686	.502		0.037	0.076	0.037	.628	−0.11; 0.19
SCL: psychoticism	.400	.128		−0.031	0.055	−0.054	.571	−0.14; 0.08

Model for SCL-90-R 2nd-order scales								
SCL: GSI	.281	.020	Age 25	1.797	1.517	0.280	.237	−1.19; 4.78
			Age 40	2.833	1.340	0.442	.035	0.20; 5.47
			Age 60	4.215	1.477	0.657	.005	1.31; 7.12
SCL: PST	.097	.064		0.021	0.030	0.095	.482	−0.04; 0.08
SCL: PSDI	.693	.011	Age 25	1.277	1.348	0.164	.344	−1.37; 3.93
			Age 40	−0.083	0.963	−0.011	.931	−1.98; 1.81
			Age 60	−1.898	1.051	−0.244	.072	−3.96; 0.17

Model for personality traits (TCI-R)								
TCI-R: novelty seeking	.571	.834		0.029	0.017	0.081	.103	−0.01; 0.06
TCI-R: harm avoidance	.007	.915	Female	0.110	0.039	0.392	.005	0.03; 0.19
			Male	0.033	0.017	0.116	.052	−0.01; 0.06
TCI-R: reward dependence	.957	.682		−0.015	0.017	−0.046	.391	−0.05; 0.02
TCI-R: persistence	.579	.897		−0.033	0.012	−0.144	.008	−0.06; −0.01
TCI-R: self-directedness	.340	.393		−0.054	0.014	−0.245	<.001	−0.08; −0.03
TCI-R: cooperativeness	.297	.468		−0.033	0.018	−0.113	.062	−0.07; 0.00
TCI-R: self-transcendence	.819	.213		0.066	0.017	0.213	<.001	0.03; 0.01

IV: independent variable. SinEff: single effects (for significant interactions). All the results adjusted by patients' sex and age.
